# Molecular identification and antiprotozoal activity of silver nanoparticles on viability of *Cryptosporidium parvum* isolated from pigeons, pigeon fanciers and water

**DOI:** 10.1038/s41598-023-30270-2

**Published:** 2023-02-22

**Authors:** Rasha M. M. Abou Elez, Amira S. A. Attia, Hala M. N. Tolba, Reham G. A. Anter, Ibrahim Elsohaby

**Affiliations:** 1 Department of Zoonoses, Faculty of Veterinary Medicine, Zagazig University, Zagazig City, Sharkia 44511 Egypt; 2 Department of Veterinary Public Health, Faculty of Veterinary Medicine, Zagazig University, Zagazig City, Sharkia 44511 Egypt; 3 Department of Avian and Rabbit Medicine, Faculty of Veterinary Medicine, Zagazig University, Zagazig City, Sharkia 44511 Egypt; 4 Department of Parasitology, Faculty of Veterinary Medicine, Zagazig University, Zagazig City, Sharkia 44511 Egypt; 5 Department of Animal Medicine, Faculty of Veterinary Medicine, Zagazig University, Zagazig City, Sharkia 44511 Egypt; 6 Department of Infectious Diseases and Public Health, Jockey Club College of Veterinary Medicine and Life Sciences, City University of Hong Kong, Hong Kong SAR, Kowloon Tong China; 7 Centre for Applied One Health Research and Policy Advice (OHRP), City University of Hong Kong, Hong Kong SAR, Kowloon Tong China

**Keywords:** Risk factors, Epidemiology, Parasitic infection

## Abstract

*Cryptosporidium* is a protozoan that causes acute gastroenteritis, abdominal pain, and diarrhea in many vertebrate species, including humans, animals and birds. A number of studies have reported the occurrence of *Cryptosporidium* in domestic pigeons. Thus, this study aimed to identify *Cryptosporidium* spp. in samples collected from domestic pigeons, pigeon fanciers, and drinking water, as well as to investigate the antiprotozoal activity of biosynthesized silver nanoparticles (AgNPs) on the viability of isolated *Cryptosporidium parvum* (*C. parvum*). Samples were collected from domestic pigeons (n = 150), pigeon fanciers (n = 50), and drinking water (n = 50) and examined for the presence of *Cryptosporidium* spp. using microscopic and molecular techniques. The antiprotozoal activity of AgNPs was then assessed both in vitro and in vivo. *Cryptosporidium* spp. was identified in 16.4% of all examined samples, with *C. parvum* identified in 5.6%. The highest frequency of isolation was from domestic pigeon, rather than from pigeon fanciers or drinking water. In domestic pigeons, there was a significant association between *Cryptosporidium* spp. positivity and pigeon's age, droppings consistency, housing, hygienic and heath conditions. However, *Cryptosporidium* spp. positivity was only significantly associated with pigeon fanciers' gender and heath condition. The viability of *C. parvum* oocysts was reduced using AgNPs at various concentrations and storage times in a descending manner. In an in vitro study, the highest reduction in *C. parvum* count was observed at the AgNPs concentration of 1000 µg/mL after a 24 h contact time, followed by the AgNPs concentration of 500 µg/mL after a 24 h contact time. However, after a 48 h contact time, a complete reduction was observed at both 1000 and 500 µg/mL concentrations. Overall, the count and viability of *C. parvum* decreased with increasing the AgNPs concentration and contact times in both the in vitro and in vivo studies. Furthermore, the *C. parvum* oocyst destruction was time-dependent and increased with increasing the contact time at various AgNPs concentrations.

## Introduction

*Cryptosporidium* spp. is an enteric parasite that causes diarrhea in humans, animals, and birds worldwide^1,2^, resulting in substantial economic losses and public health risk^3^. The prevalence of *Cryptosporidium* infection in humans, animals and poultry varies^4,5^, with symptoms ranging from mild to severe diarrhea or asymptomatic illness, depending on the immune status of the infected host^6^. There are more than 26 *Cryptosporidium* spp., with *Cryptosporidium parvum* (*C. parvum*) being the most common zoonotic species that affects humans, animals, and birds^7^. The fecal–oral route transmits cryptosporidiosis, either directly through contact with infected humans, animals, and birds or indirectly through contaminated water or food with a low infectious dose of 10 oocysts^2^.

Many birds, including chickens, turkeys, geese, ducks, pigeons, lovebirds, cockatiels, and ostriches, are known to be biological reservoirs of *Cryptosporidium* spp., particularly *C. parvum*; and can transmit infection to humans and animals^1^. Cryptosporidiosis in birds is characterized by respiratory and intestinal symptoms^8^ and can be spread between birds through the ingestion of oocysts in contaminated environments^9^. Furthermore, contaminated drinking water with *Cryptosporidium* oocysts is a major source of infection for humans and livestock, resulting in millions of deaths each year^10^. *Cryptosporidium* is able to pass through filtered or unfiltered drinking water systems due to the small size of its oocysts^11^. Additionally, groundwater supplies can be polluted by the infiltration of contaminated surface waters^12^.

The diagnosis of *Cryptosporidium* infection is typically based on microscopic detection of oocysts in fecal, stool, and water samples. However, this method has low sensitivity for determining *Cryptosporidium* spp.^13^. Polymerase chain reaction (PCR) is considered the most popular and sensitive method to differentiate between *Cryptosporidium* spp.^14^. Immunomagnetic separation (IMS) is also commonly used method for purifying oocysts before DNA extraction, as it effectively eliminates or greatly reduces any inhibitory substances that could interfere with PCR amplification^15^.

Several factors make *Cryptosporidium* inactivation difficult in both developed and developing countries including: the high survival rate of *Cryptosporidium* oocyst in water for more than two years at 20 °C, the resistance to most disinfections such as ultraviolet irradiation, hypochlorous acid, and chloramine^16,17^, low host specificity^18^ and low infectious dose (10–132 oocysts)^19^. Thereby, biosynthesized silver nanoparticles (AgNPs) are used as a new alternative for combating *C. parvum* oocysts by liberating silver ions (Ag+), which cause oxidative stress via the release of reactive oxygen species^20,21^. The released Ag + ions and nanoparticles can demolish the sporozoites and destruct the oocyst cell wall^22^. Although the exact mechanism of action of AgNPs against protozoa is not yet fully understood, several studies have documented their antiprotozoal effect on other protozoa, such as *Leishmania*^23,24^.

This study was designed to (1) determine the frequency of *C. parvum* infection in domestic pigeons, pigeon fanciers, and drinking water in Sharkia Governorate, Egypt, (2) investigate the potential risk factors associated with *C. parvum* infection, and (3) explore the antiprotozoal effect of different concentrations and contact times of biosynthesized AgNPs on *C. parvum* oocyst count and viability.

## Materials and methods

### Ethical statement

This study was reviewed and approved by the Institutional Animal Care and Use Committee (IACUC) of Zagazig University (Ref. No.: ZU-IACUC/2/F/211/2022). All procedures involving animals were performed in accordance with the ARRIVE criteria. However, procedures involving human participants were performed in accordance with the 1964 Declaration of Helsinki and its later amendments or comparable ethical standards. In addition, written informed consent was obtained from the fanciers for the participation in this study.

### Sample collection

Between September 2021 and March 2022, 150 fresh fecal samples were collected from domestic pigeons (*Columba livia domestica*). The pigeons were randomly selected from various households (each with an average of 100–300 pigeons) in Sharkia Governorate, Egypt. Twenty pigeons were sampled randomly from each household, and each sample consisted of 4–5 fecal deposits. Additionally, samples of stool (n = 50) and drinking water (n = 50) were collected from pigeon fanciers and water sources intended for pigeon and human drinking. Twenty-five liters of water samples were collected in sterile plastic polypropylene containers. All samples were labelled with identification numbers and dates before being sent to the laboratory for further analysis.

Data were collected on the sampled pigeons (including sex, age, food type, hygienic condition, location, fecal consistency, and health status), pigeon fanciers (including age, sex, education level, stool consistency, health condition, knowledge about disease epidemiology, and source of drinking water), and water source types (e.g., surface and underground water).

### Microscopical examination

Fecal and stool samples were examined using a wet preparation method (WM) followed by the Sheather’s sugar flotation technique. This technique uses a sucrose solution with a specific gravity of 1.21 to concentrate the sample. Once the sample is concentrated, thin smears were made on glass slides. The slides were air-dried, methanol-fixed, stained with modified Ziehl–Neelsen (MZN) staining, and examined under a light microscope (40X, 100X).

To examine the water samples, 5 L of each sample was filtered using a 142-mm diameter membrane filter with a pore size of 1.2 μm using a vacuum pump. The filtered sample was centrifuged for 10 min at 3000 g. The sediment pellet containing oocysts was subjected to sucrose flotation and immunomagnetic separation (IMS) and PCR methods^25^. One drop of this concentrate was smeared on a slide, stained using the modified Ziehl–Neelsen technique^26^, and then examined under a microscope (40X, 100X). Microscopically positive samples were stored at − 20 °C for subsequent DNA extraction. The remaining microscopically positive samples were stored in refrigerator in order to evaluate antiprotozoal activity of nanoparticles.

### Molecular identification

Frozen *Cryptosporidium*-positive fecal, stool and water samples were further purified by IMS using the Dynabead anti-*Cryptosporidium* kit (Dynal Inc, Lake Success, NY, USA) following the manufacturer’s recommended procedures. The IMS-purified oocysts were subsequently captured using a magnetic device (MPC; Dynal Inc, Lake Success, NY, USA) and subjected to five freeze–thaw cycles (− 70 °C for 30 min and 56 °C for 30 min). The QIAamp DNA Mini Kit (QIAGEN GmbH, Hilden, Germany) was used for DNA extraction from the purified *Cryptosporidium* oocysts according to the manufacturer’s guidelines^15^. The extracted DNA was used for the amplification of the 18S rRNA gene as previously described^27^. The isolates identified as *Cryptosporidium* spp. were then subjected to molecular identification of the *Actin* gene (400 bp) for *C. parvum*^28^. A reaction mixture without DNA was used as a negative control and *Cryptosporidium* spp. and *C. parvum* were served as positive controls. The PCR products were detected on 2% agarose gel which stained by ethidium bromide.

### Antiprotozoal activity of biosynthesized silver nanoparticles

#### Collection and purification of oocysts

*C. parvum* oocysts were isolated and purified from heavily infected samples as previously described^29^. Samples were examined using the modified MZN technique. Samples containing more than 4–5 *C. parvum* oocysts per field were selected for analysis and filtered through four layers of gauze. The preserved oocysts were kept for three days at 4 °C before the purification process. The ethyl acetate sedimentation technique (with phosphate-buffered saline [PBS] replacing formalin) was used for oocyst purification, followed by discontinuous sucrose flotation^30,31^. Potassium dichromate (1:4, v/v) was used to preserve the oocysts at 4 °C for subsequent experiments.

#### In vitro exposure to biosynthesized AgNPs

A previously prepared AgNPs from *Aspergillus niveus*, accession number MT319815, were used^32^. AgNPs were oval, cubic and rod-shaped, with uniformly distributed at 6.49 nm and an absorption peak at 420 nm. The AgNPs had a particle size of 27 nm with various functional groups and a negative charge of 30.4 mv. The Neubauer hemocytometer was used to determine the concentration of *C. parvum* oocysts. The mean of the four hemocytometer counts of the stock suspension with the dilution factor was calculated after several washes with PBS (to remove potassium dichromate)^33^. A 10-μL aliquot of oocyst suspension was pipetted between the counting chambers and hemocytometer cover slides and enumerated under a 40X objective using a phase-contrast microscope. The oocysts with a final concentration of 1 × 10^3^/mL were then incubated in sterile PBS (pH 7.4) and an antibiotic (Pen/Strep/Amphotericin B (100 ×), BioWhittaker®, Lonza) suspension (10,000-U Pen/mL, 10,000-μg Strep/mL, and 25-μg Amphotericin B/mL) at room temperature. The purified oocysts were suspended in normal saline and stored at 4 °C for a maximum of 3 days before use. Various concentrations of AgNPs (i.e., 10, 50, 500, and 1000 μg/mL) were added to the calculated oocysts’ suspension to form a total volume of 1 mL.

The count and viability of the treated *C. parvum* oocysts were determined after 3, 6, 12, 24, and 48 h of incubation with AgNPs. The remaining settled oocysts were then gently mixed for 15 min with 2 mL 0.1% eosin stain to detect viability. Viable oocysts were unstained while nonviable oocysts were stained red. *C. parvum*-positive oocysts were counted before and after exposure to AgNPs according to Suresh and Rehg^34^, using a hemocytometer slide under a bright-field microscopy, with the use of the following equation:

$${\text{C}} = \left( {{\text{T}}.{\text{ D}}} \right)/{\text{W}}$$

where C = oocyst count, T = the total number of counted oocysts, D = dilution factor, and W = the volume of tested water sample in (mL).

The minimum lethal concentration (MLC) was the lowest AgNPs concentration leading to the absence of viable *C. parvum* oocysts^35^. The test was repeated three times for each AgNPs concentration and each sample.

#### In vivo infectivity assays

Pathogen-free Swiss Albino mice with an average weight of 27–34 g and aged nine weeks were used in this study. The mice were divided into five groups labelled G1, G2, G3, G4, and G5. Each group contained three mice, and all mice were kept separately in standard conditions. Group G1 was the control group and received *C. parvum* oocysts without AgNPs. Groups G2, G3, G4, and G5 received *C. parvum* oocysts treated with different concentrations of AgNPs at different contact times (3, 6, 12, 24, and 48 h).

The mice received different concentrations of AgNPs suspension, and the viable oocysts were counted using a hemocytometer^36^. All mice gavaged with 1000 *C. parvum* oocysts diluted in 75 μL distilled water with or without AgNPs exposure at the same time. Fresh fecal pellets were individually collected from each mice by applying gentle pressure on their abdomens over the experimental period (i.e., 2, 4, 6, 8, and 10 days), while counting *Cryptosporidium* oocysts. All mice were euthanized 10 days after being inoculated. *C. parvum* infection were then classified as positive or negative for each group based on microscopic examination and molecular identification.

### Statistical analysis

The association between *Cryptosporidium* spp. positivity and potential risk factors was evaluated using Chi-square and Fisher's exact tests. A two-way analysis of variance was used to identify the variations in AgNPs concentrations and *Cryptosporidium* oocysts at different contact times. Statistical analysis was performed using STATA version 16 (Stata Corp., College Station, TX) and a *P*-value < 0.5 was considered statistically significant.

## Results

### *Cryptosporidium* spp. isolation and identification

**Figure 1:**
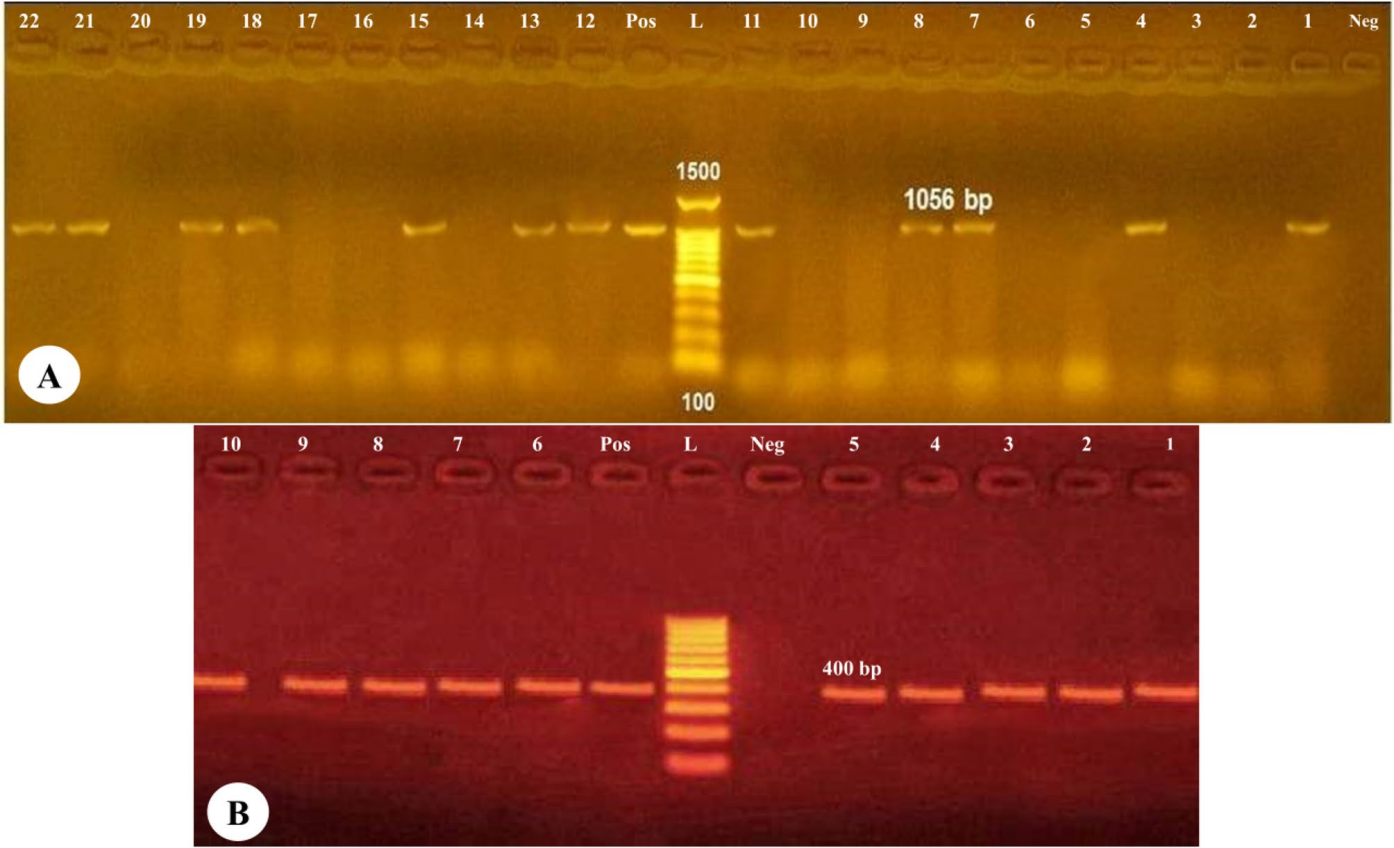
Agarose gel electrophoresis for the products of PCR fingerprinting of (**A**) *Cryptosporidium* spp. and (**B**) *C. parvum*. Lane (L): DNA marker ladder; Lane Pos: positive control and Lane Neg: negative control.

*Cryptosporidium* spp. were molecularly identified in 39 (16.9%) of all samples examined and mostly identified in samples from domestic pigeon (n = 29; 19.3%), followed by pigeon fanciers (n = 8; 16%) and water samples (n = 2; 6.7%) (Fig. 1A). *C. parvum* was molecularly detected in 12 (5.2%) of all samples, including 7 (4.7%) in domestic pigeon, 3 (6%) in pigeon fanciers, and 2 (6.7%) in drinking surface water supplies (Fig. 1B).

Table 1 shows significant association between *Cryptosporidium* spp. positivity and pigeon's age, droppings consistency, housing, hygienic and heath conditions. However, *Cryptosporidium* spp. positivity was only significantly associated with pigeon fanciers' gender and health condition.

**Table 1:** Proportion and risk factors associated with *Cryptosporidium* spp. infection in domestic pigeon, pigeon fanciers and tape water.

Sample source	Risk factors		No. of tested samples	No. positive *Cryptosporidium* spp. (%)	No. positive *C. parvum* (%)	*P*-value
Pigeon (n = 150)	Age	< 1y	60	20 (33.3)	5 (8.3)	0.000
		≥ 1y	90	9 (10.0)	2 (2.2)	
	Sex	Female	80	10 (12.5)	2 (2.5)	0.252
		Male	70	19 (27.1)	5 (7.1)	
	Droppings consistency	Toothpaste	101	6 (5.9)	0	0.000
		Watery	49	23 (46.9)	7 (14.3)	
	Housing	Multiple pens	41	23 (56.1)	7 (17.1)	0.000
		Nest boxes	50	3 (6.0)	0	
		Perches	59	3 (5.1)	0	
	Hygienic conditions	Good	99	7 (7.1)	0	0.000
		Bad	51	22 (43.1)	7 (13.7)	
	Health conditions	Healthy	90	4 (4.4)	0	0.000
		Diseased	60	25 (41.7)	7 (11.7)	
	Living area	Rural	91	19 (20.9)	5 (5.5)	0.705
		Urban	59	10 (16.9)	2 (3.4)	
Pigeon fanciers (n = 50)	Age	≤ 30	30	6 (20.0)	3 (10.0)	0.450
		> 30	20	2 (10.0)	0	
	Gender	Male	40	6 (15.0)	3 (7.5)	0.000
		Female	10	2 (20.0)	0	
	Group health	Healthy	40	1 (2.5)	0	0.000
		Diseased	10	7 (70.0)	3 (30.0)	
	Level of education	High	20	0	0	–
		Low	30	8 (26.7)	3 (10.0)	
	Stool consistency	Normal	40	0	0	–
		Watery	10	8 (80.0)	3 (30.0)	
	Knowledge about disease transmission	Yes	10	0	0	–
		No	40	8 (20.0)	3 (7.5)	
	Drinking water types	Tape	30	8 (26.7)	3 (10.0)	–
		Filtered	20	0	0	
Tape water (n = 30)	Resources	Underground	17	0	0	–
		Surface	13	2 (15.4)	2 (15.4)	
Total		230		39 (16.9)	12 (5.2)	

### Antiprotozoal activity of biosynthesized AgNPs

#### In vitro exposure to biosynthesized AgNPs

**Figure 2:**
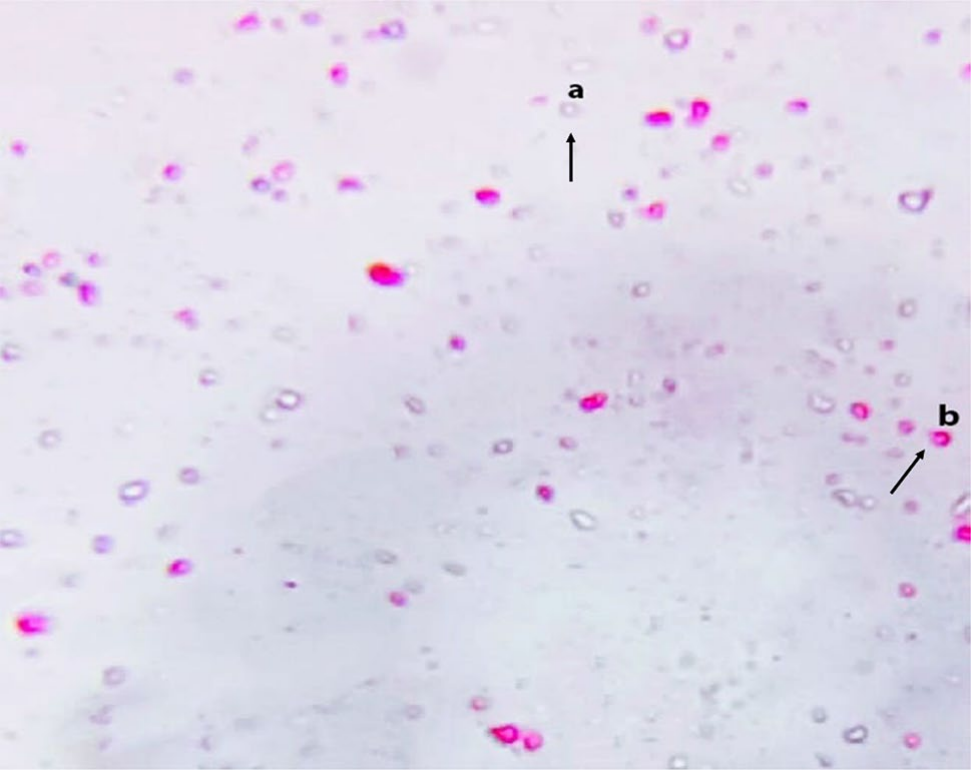
*C. parvum* oocysts treated with AgNPs and stained with 0.1% eosin dye (**a**) viable oocysts (unstained) and (**b**) inviable oocysts (stained red) at X 40.

**Figure 3:**
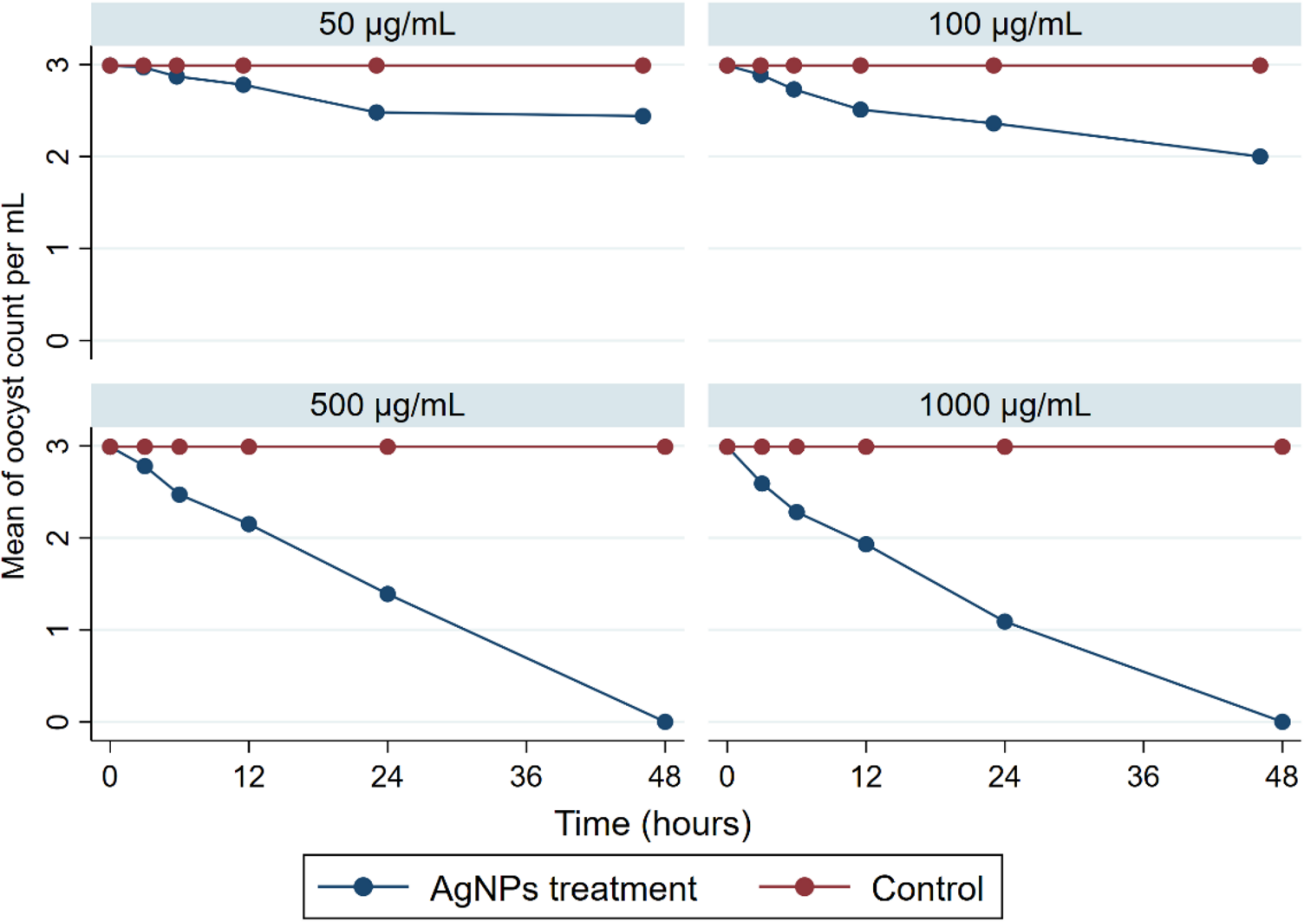
Time kill curve of *C. parvum* treated with AgNPs at different concentrations.

The antiprotozoal activity of the biosynthesized AgNPs on the viability of *C. parvum* oocysts was tested. The results showed that the AgNPs had a significant inhibitory effect on the viability of *C. parvum* oocysts (Fig. 2). In addition, the effects of storage time on *C. parvum* treated with biosynthesized AgNPs at different concentrations (i.e., 50, 100, 500, and 1000 µg) were evaluated. Figure 3 illustrates the total count of *C. parvum* oocysts in the treated samples and the control group at different contact times. A nonsignificant difference in the control samples at different storage times was observed.

The total count of *C. parvum* oocysts treated with AgNPs at different concentrations in the control group at 0 h was 2.99 ± 0.01. A significant decrease in *C. parvum* count was observed after 3 h, 6 h, 12 h and 24 h of storage at concentrations of 500 µg/mL and 1000 µg/mL ((2.77 ± 0.007 and 2.59 ± 0.03), (2.46 ± 0.007 and 2.27 ± 0.02), (2.15 ± 0.03 and 1.92 ± 0.02), and (1.39 ± 0.08 and 1.08 ± 0.08), respectively), whereas no oocysts were detected after 48 h of storage. The time-kill curve for *C. parvum* treated with AgNPs at different concentrations gradually decreased as the storage time increased, with the MLC of AgNPs being at 500 µg/mL in this study.

#### In vivo infectivity assays

Table 2 shows that mice in group G3, G4 and G5 infected with *C. parvum* oocysts treated with AgNPs at different concentrations and storage times had significantly lower *C. parvum* oocyst counts than those in the control group (G1) and G2. The groups infected with *C. parvum* exposed to AgNPs at the concentration of 500 µg/mL for 48 h and 1000 µg/mL for 48 h showed no infection, with a 0/3 infection rate.

**Table 2:** Count and infection rate of *C. parvum* treated with different concentrations of AgNPs in laboratory mice.

Groups (AgNPs concentration µg/mL)	Contact time (hours)	Infection rate^a^	Viable *C. parvum* oocyst count^b^
G1 (control)	3	3/3	3.39 ± 0.004
	6	3/3	3.39 ± 0.004
	12	3/3	3.39 ± 0.004
	24	3/3	3.39 ± 0.004
	48	3/3	3.39 ± 0.004
G2 (50)	3	3/3	3.34 ± 0.009
	6	3/3	3.29 ± 0.005
	12	3/3	3.19 ± 0.01
	24	3/3	2.98 ± 0.01
	48	3/3	2.84 ± 0.02
G3 (100)	3	3/3	3.29 ± 0.004
	6	3/3	3.18 ± 0.01
	12	3/3	3.1 ± 0.008
	24	3/3	2.89 ± 0.02
	48	3/3	2.75 ± 0.02
G4 (500)	3	3/3	3.15 ± 0.007
	6	3/3	2.89 ± 0.004
	12	3/3	2.33 ± 0.05
	24	3/3	1.65 ± 0.04
	48	0/3	0
G5 (1000)	3	3/3	2.96 ± 0.005
	6	3/3	2.59 ± 0.008
	12	3/3	1.79 ± 0.01
	24	3/3	1.35 ± 0.04
	48	0/3	0.0

^a^No. of positive mice/no. of inoculated mice.

^b^Each value represents the mean ± standard deviations of three counts of viable oocysts.

## Discussion

*Cryptosporidium* is a zoonotic disease that is capable of causing serious public health risks. Several studies have found that domestic pigeons can act as reservoir host for *Cryptosporidium*, with direct contact between pigeons and fanciers creating favorable conditions for transmission^37,38^. In current study, *Cryptosporidium* spp. was identified in 19.3% of pigeon samples with 4.7% of these samples being *C. parvum*. A similar rate (5.9%) was reported in pigeons in Spain^38^, while a lower rate (0.82%) was reported in China^4^. However, higher rates of *Cryptosporidium* infection in pigeons were found in Bangladesh (50%)^39^ and Egypt (46.2%)^40^.

In this study, we assessed number of disease determinants that might be associated with *Cryptosporidium* spp. infection in domestic pigeons. Results showed that there is significant association between infection and pigeon age, housing, hygienic and health conditions. Previous studies have reported that *Cryptosporidium* infection were more frequent in young pigeons than adults^37,41^. The undeveloped immune system in young birds might be the main cause for increasing susceptibility of young birds than adults^7^. Unhygienic conditions in cages and high stocking density were also identified as risk factors for *Cryptosporidium* infection^9^. These findings suggest that it is important to ensure that pigeons have adequate housing and hygiene conditions in order to reduce the risk of infection^42^.

Regarding pigeon fanciers, *Cryptosporidium* spp. was detected in 16% of the sample tested in this study, of which 6% were identified as *C. parvum.* In addition, a strong association was found between *Cryptosporidium* infection and fanciers’ gender and health condition. Previous studies in human cryptosporidiosis have reported similar isolation rates. For example, Chen, et al.^43^, found *C. parvum* infection in 6.9% of tested children in China. Additionally, several studies have found that immunocompromised individuals have high prevalence of cryptosporidiosis than healthy individuals^44,45^. In this study, all infections were reported in fanciers with low education level, which is in agreement with the findings of Elshahawy and AbouElenien^46^ who found that individules without formal education are more likely to be infected with cryptosporidiosis than those with formal education.

Regarding drinking water sources, *Cryptosporidium* spp. has been isolated from surface water only, which is in agreement with the findings of Stokdyk, et al.^47^, who found that groundwater was free of *Cryptosporidium*. Simmons III, et al.^48^ have also detected *Cryptosporidium* oocytes in surface water samples in North Carolina streams and reservoirs used as sources of drinking water. Other studies have reported *Cryptosporidium* oocytes in both surface and underground water supplies, but the rate was higher in surface water^46,49,50^.

Because of the side effects and cost of chemical drugs for water treatment, particularly in the rural areas, the use of natural products for water treatment is needed. In recent years, developing countries have begun to use nanotechnology as an antiprotozoal and antimicrobial^51–53^. Since *Cryptosporidium* oocysts remain viable in water for up to a year, even in purified drinking water and cause epidemics^54^. In this study, the antiprotozoal activity of AgNPs against *C. parvum* oocysts in vitro, and the obtained results were confirmed in an in vivo infectivity assay.

The antiprotozoal activity of AgNPs has been documented in different studies^55,56^. The findings from the present study are in line with those from previous studies which have found that AgNPs can have a significant inhibitory effect on the viability of *C. parvum* oocysts^22^. The highest reduction in count and viability of the *C. parvum* was observed at the AgNPs concentration of 500 µg/mL and 1000 µg/mL, while the least reduction was noted at the 50 µg/mL concentration. Similar results were obtained by Cameron, et al.^22^, who have reported that AgNPs at a concentration of 500 μg/mL significantly reduced the viability of *C. parvum* oocysts. Furthermore, Saad, et al.^57^ have found that *C. parvum* oocysts isolated from human and animal feces became inactive after nanoparticle treatment. The inhibitory effects of AgNPs on *C. parvum* oocysts may be attributed to their small size, which cause them directly diffuse into the cell membrane pores, and cause toxic effects on protozoal cells^58^. Moreover, Iavicoli, et al.^59^ have found that AgNPs with smaller diameters seem to be more effective than larger ones.

The MLC of AgNPs in this study was determined to be at a concentration of 500 µg/mL. However, Ahmed, et al.^29^ reported that 3000 μg/mL was the MLC of Chitosan nanoparticles to kill more than 90% of oocysts.* Cryptosporidium* had a hard cell wall; so it requires longer contact times with nanoparticles for effective destruction. Similarly, a previous study on the oocyst of *Cyclospora cayetanensis* required a longer contact time with magnesium nanoparticles to be destructed^60^. On the other hand, Hassan, et al.^61^ reported that shorter contact times are recommended for better destruction of *C. parvum* oocysts (i.e., 30 min at 1 ppm or 1 h at 0.1 ppm concentrations of AgNPs). The difference between our results and other studies could be attributed to the sample type, methodology, and properties of the used nanoparticles.

## Conclusions

*Cryptosporidium* spp. and *C. parvum* were found in Egyptian domestic pigeons, pigeon fanciers, and drinking water, posing a public health risk. Understanding the transmission mode of *Cryptosporidium* infection is essential, and proper hygiene and sanitation practices should be implemented. This study provided evidence of the safety and efficacy of biosynthesized AgNPs with reasonable contact times as a new nanoform agent in treating and controlling *C. parvum* infection in domestic pigeons in farms, drinking water in large-scale water plants, and humans.

## Data Availability

All data generated or analyzed during this study are included in this published article.
